# Unveiling the Impact of Climatic Factors on the Distribution Patterns of *Caragana* spp. in China’s Three Northern Regions

**DOI:** 10.3390/plants14152368

**Published:** 2025-08-01

**Authors:** Weiwei Zhao, Yujia Liu, Yanxia Li, Chunjing Zou, Hideyuki Shimizu

**Affiliations:** 1 College of Life Science & Technology, Xinjiang University, Urumqi 830017, China; 107552301038@stu.xju.edu.cn (W.Z.); 13399766117@163.com (Y.L.); 2 School of Life Science, East China Normal University, Shanghai 200241, China; 3 National Institute for Environmental Studies, 16-2 Onogawa, Tsukuba 305-8506, Ibaraki, Japan; hideshimizu@hotmail.com

**Keywords:** genus *Caragana*, distribution pattern, Biomod2 model, moisture-temperature indexes, China’s three northern regions

## Abstract

Understanding the impacts of climate change on species’ geographic distributions is fundamental for biodiversity conservation and resource management. As a key plant group for ecological restoration and windbreak and sand fixation in arid and semi-arid ares in China’s Three Northern Regions (Northeast, North, and Northwest China), *Caragana* spp. exhibit distribution patterns whose regulatory mechanisms by environmental factors remain unclear, with a long-term lack of climatic explanations influencing their spatial distribution. This study integrated 2373 occurrence records of 44 *Caragana* species in China’s Three Northern Regions with four major environmental variable categories. Using the Biomod2 ensemble model, current and future climate scenario-based suitable habitats for *Caragana* spp. were predicted. This study innovatively combined quantitative analyses with Kira’s thermal indexes (warmth index, coldness index) and Wenduo Xu’s humidity index (HI) to elucidate species-specific relationships between distribution patterns and hydrothermal climatic constraints. The main results showed that (1) compared to other environmental factors, climate is the key factor affecting the distribution of *Caragana* spp. (2) The current distribution centroid of *Caragana* spp. is located in Alxa Left Banner, Inner Mongolia. In future scenarios, the majority of centroids will shift toward lower latitudes. (3) The suitable habitats for *Caragana* spp. will expand overall under future climate scenarios. High-stress scenarios exhibit greater spatial changes than low-stress scenarios. (4) Hydrothermal requirements varied significantly among species in China’s Three Northern Regions, and 44 *Caragana* species can be classified into five distinct types based on warmth index (WI) and humidity index (HI). The research findings will provide critical practical guidance for ecological initiatives such as the Three-North Shelterbelt Program and the restoration and management of degraded ecosystems in arid and semi-arid regions under global climate change.

## 1. Introduction

Global climate change is reshaping the biogeographic distribution pattern with unprecedented speed and intensity [1]. The Intergovernmental Panel on Climate Change (IPCC) Sixth Assessment Report has unequivocally pointed out that the surface warming rate over the past 50 years has exceeded all observed levels in the past two millennia, accompanied by intensified spatiotemporal heterogeneity in precipitation regimes [2,3]. Such drastic climatic shifts are triggering range shifts, contractions, or expansions of species distributions. As one of the most climate-sensitive zones globally, arid and semi-arid regions exhibit vegetation dynamics that not only determine regional ecological security but are also crucially linked to global sustainability priorities, including carbon sequestration potential and land degradation mitigation under the United Nations Sustainable Development Goals (SDGs) [4]. Therefore, projecting shifts in plant habitat suitability across arid and semi-arid regions under climate change is critical for species conservation efforts and ecosystem restoration initiatives.

China’s Three Northern Regions (Northeast, North, and Northwest China), as the world’s largest mid-latitude arid zone, feature particularly fragile ecosystems and exhibit a significantly stronger warming trend than the global average. Desertified land in China’s Three Northern Regions accounts for approximately 27% of China’s total land area [5] and exhibits a noticeable expansion trend. Spanning 13 provincial-level administrative units across northern China, this region features complex and diverse geographic environments and climatic conditions. Its striking longitudinal vegetation gradient transitions from coastal forest belts through central grasslands to inland desert ecosystems. This region sustains the planet’s largest afforestation project (Three-North Shelterbelt Program) while functioning as the primary source area for Asian dust storms [6]. This ecologically fragile zone harbors approximately 75% of China’s total *Caragana* Far., which are widely distributed across the region. Owing to their exceptional drought resistance, nitrogen-fixing capacity, and dual functions in windbreak and sand stabilization, *Caragana* has become a keystone taxon for restoring degraded ecosystems in these areas. *Caragana* species are extensively utilized in China’s Three-North Shelterbelt Program for sand control projects, playing a crucial role in restoring ecosystems in arid regions. For instance, *C. korshinskii* and *C. microphylla* serve as the dominant sand-fixation species in China’s desert, semi-desert, and loess areas. However, despite the acknowledged ecological significance of the *Caragana* spp., research gaps persist in understanding the environmental drivers shaping their distribution patterns [7], particularly their spatial dynamics in China’s Three Northern Regions. This critical knowledge deficit severely impedes science-based optimization of ecological restoration strategies under accelerating climate change.

In recent years, Species Distribution Models (SDMs) have emerged as pivotal tools for deciphering plant–environment relationships in biogeography. By integrating species occurrence data with environmental variables through statistical and machine learning algorithms, these models enable robust predictions of potential species distribution patterns [8,9]. Currently, widely used SDMs include the Maximum Entropy Model (MaxEnt), Random Forest (RF), Generalized Boosted Regression Models (GBM), and Generalized Linear Model (GLM), among others [10,11]. However, these models exhibit inherent strengths and limitations in predicting species distributions. The Biomod2 ensemble model can integrate these high-performing single models to generate predictions. Through consensus forecasting, Biomod2 achieves enhanced stability and accuracy compared to single-model approaches, thus providing more reliable projections for conservation planning [12]. Since its release in 2003, the Biomod2 ensemble model has gained widespread recognition and application [13]. In arid and semi-arid regions, SDMs have been successfully applied to predict the distribution and assess climate change responses of leguminous plants. For instance, SDM projections indicated that climate change will cause a contraction in the habitat range of *Pterocarpus erinaceus* distributed in arid zones [14]. However, existing studies on *Caragana* distribution have predominantly relied on individual environmental variables and single-model approaches, lacking systematic integration of multi-model synergies and cross-factor analyses.

Previous studies have shown that *Caragana* species exhibit a gradient distribution pattern in China’s Three Northern Regions [15]. From *Caragana microphylla* in the northeastern Songnen Plain to *C. korshinskii* in the northwestern desert zones, their habitats span an east–west bioclimatic transect. This broad-scale biogeographic configuration implies that different species likely occupy partitioned ecological niches through divergent hydrothermal adaptation strategies [16]. However, conventional studies predominantly focus on ecological adaptation mechanisms of individual species [17], such as the deep-rooting drought resistance mechanisms in *C. korshinskii* [18] or the soil-ameliorating effects of *C. sinica* [19]. These approaches lack systematic investigations at the genus level to disentangle the climatic drivers shaping distribution patterns across the *Caragana* species. Crucially, the regulatory effects of climatic factors on plant distributions exhibit multidimensional complexity [20]. In thermal dimensions, Kira’s warmth index (WI) and coldness index (CI) quantify thermal constraints on plant distributions through accumulated temperature thresholds, having demonstrated robust efficacy in delineating East Asian vegetation zones. In hydrological dimensions [21,22], Wenduo Xu’s humidity index (HI), formulated based on vegetation–climate interactions and precipitation–temperature synchrony, calculates the ratio of annual precipitation to WI [23]. These indexes directly capture vegetation responses to coupled hydrothermal influences, providing a quantitative framework to unravel species–climate relationships [24].

Focusing on the above issues, we selected the Three Northern Regions of China (spanning 13 provinces, autonomous regions, and municipalities) as the study area, with *Caragana* spp. as the target species. We employed the Biomod2 ensemble model to integrate multiple algorithms, aiming to uncover the relationships between geographic distribution patterns of *Caragana* spp. and environmental drivers across this regional-scale arid/semi-arid biome. Innovatively, we applied quantitative methods, introducing Kira–Xu’s moisture–temperature indexes (WI, HI) to provide a climatological explanation for the ecogeographic distribution patterns of *Caragana* species in China’s Three Northern Regions. Objectives of this study were as follows: (1) to determine the environmental factors that dominate the geographical distribution pattern of the genus in the current context; (2) to project habitat suitability shifts for the genus under future climate scenarios; (3) to evaluate the species-specific hydrothermal threshold of *Caragana* species. The research findings will provide a theoretical foundation for biodiversity conservation of the *Caragana* species, ecological barrier construction in China’s Three Northern Regions, restoration of degraded vegetation, and climate change adaptive management, while also offering a paradigmatic reference for studying phytogeographic distribution mechanisms in arid and semi-arid regions [25,26].

## 2. Materials and Methods

### 2.1. Acquisition and Screening of Species Distribution Data

**Figure 1:**
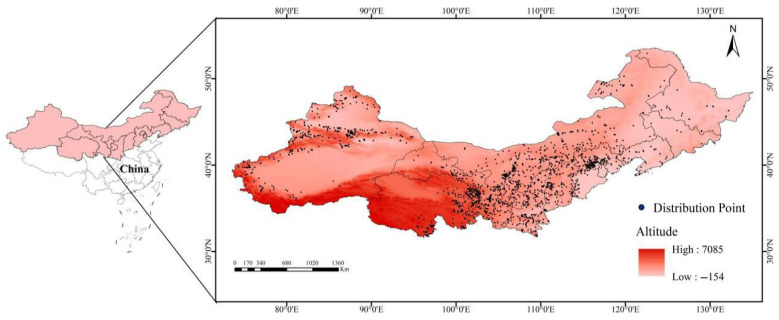
Geographic distribution records of *Caragana* spp. in China’s Three Northern Regions.

The distribution data of *Caragana* species across 13 provincial-level administrative regions in China’s Three Northern Regions were systematically compiled from three principal sources [27]: Global Biodiversity Information Facility (GBIF, https://www.gbif.org, accessed on 21 November 2024, DOIs are provided in the Supplementary Materials), Chinese Virtual Herbarium (CVH, https://www.cvh.ac.cn/, accessed on 21 November 2024), and National Specimen Information Infrastructure (NSII, http://www.nsii.org.cn/, accessed on 21 November 2024). All *Caragana* species were treated uniformly, with synonymous or misidentified records removed through comparative verification. To avoid model overfitting, spatial filtering was performed using the software of ENMTools.pl to remove redundant occurrence records, retaining only one occurrence per 2.5 arc-minute grid cell. This process resulted in 2373 validated distribution records for *Caragana* spp. (Figure 1).

### 2.2. Acquisition and Screening of Environmental Variables for Model Construction

In this study, we selected 24 natural environmental variables and 1 anthropogenic variable to construct SDMs. Natural environmental variables included 19 bioclimatic variables (bio1–bio19) from WorldClim version 2.1 (http://www.worldclim.org/), 4 commonly used topsoil variables from the National Cryosphere Desert Data Center (http://www.ncdc.ac.cn), and terrain elevation data from the Geospatial Data Cloud (https://www.gscloud.cn/). The selection of topsoil and elevation data was based on the biological characteristics of *Caragana* species and the environmental features of China’s Three Northern Regions. Available water content class determines drought stress responses, topsoil pH influences nutrient availability and rhizobial nitrogen fixation, while topsoil organic carbon and texture classification influence fertility and root development. Altitude serves as the primary topographic variable for analyzing species distribution at regional scales. The anthropogenic variable was the Human Footprint Index obtained from SEDAC (http://sedac.ciesin.columbia.edu/wildareas/, accessed on 19 March 2025). The future climatic variables were derived from the BCC-CSM2-MR climate model under three Shared Socio-Economic Pathways (SSPs) for the periods of 2021–2040 (2030s) and 2041–2050 (2050s): SSP126 (low-stress scenario), SSP370 (moderate-stress scenario), and SSP585 (high-stress scenario). To maintain the comparability of models across time series, this study assumes that other environmental variables, except climate, remain constant in future distribution projections. Based on the coarsest data layer, the aforementioned data is based on the study area scope and clipped to a 2.5 arc-minute resolution using mask extraction in ArcGIS 10.8 [28].

To ensure model validity and avoid overfitting due to high multicollinearity among environmental variables, we conducted Pearson correlation tests and Variance Inflation Factor (VIF) tests using the usdm package in R software (R 4.3.3). Variables with pairwise correlation coefficients |r| < 0.7 and VIF < 5 were retained [29,30], resulting in the selection of 10 environmental variables for subsequent model construction (Table 1).

### 2.3. Ensemble Model Construction and Accuracy Evaluation

The ‘Biomod2’ ensemble modeling platform in R (4.3.3) was used to simulate the suitable distribution range of *Caragana* for current and future periods (2030s, 2050s). To overcome methodological constraints of single-algorithm approaches, we employed a consensus forecasting framework incorporating eight algorithms: Random Forest (RF), eXtreme gradient boosting (XGBoost), Classification Tree Analysis (CTA), Flexible Discriminant Analysis (FDA), Generalized Additive Model (GAM), Generalized Boosting Model (GBM), Generalized Linear Models (GLM), and Maximum Entropy Models (MaxEnt).

During the construction of single models, default parameters were applied for all algorithms. To enhance the accuracy of simulations and reduce random bias, we generated two sets of pseudo-absence points (8000 points per set). To assess the average predictive performance of the ensemble model, we randomly employed 80% of the sample data as the training set and reserved 20% as the validation set. Each algorithm was executed ten times.

For model evaluation, the True Skill Statistic (TSS) and Area Under the Receiver Operating Characteristic Curve (AUC) were used to assess model accuracy. The AUC ranges from 0.5 to 1, and the TSS ranges from −1 to 1, with values closer to 1 indicating better model performance. Generally, AUC values above 0.8 indicate good predictive performance, while values below 0.7 suggest bad performance [31]. TSS values exceeding 0.55 are considered indicative of medium predictive accuracy [32,33]. Based on single-model evaluations, models with TSS values greater than 0.55 were selected to construct an ensemble model using the weighted average method (WM).

### 2.4. Suitable Habitats Change and Centroid Migration

Based on the ensemble model outputs, we conducted a reclassification in ArcGIS 10.8 to categorize *Caragana* habitat suitability into four classes according to distribution probability [34]: non-suitable areas (<0.2), minimally suitable areas (0.2–0.4), moderately suitable areas (0.4–0.6), and highly suitable areas (>0.6). Higher category levels indicate areas suitable for a greater number of *Caragana* species. Additionally, Raster reclassification was used to calculate the territory area corresponding to each category. Areas with species presence probability > 0.2 were classified as potential distribution areas. Then we utilized the ArcGIS 10.8 plugin SDMtoolbox tool to calculate the centroid positions and migration trends of the potential distribution of *Caragana* simulated by different occurrence data sources during the current period and the 2030s and 2050s periods.

### 2.5. Acquisition and Calculation of Moisture–Temperature Indexes for Different Caragana Species

The meteorological data for calculating Kira’s thermal indexes (WI, CI) and Xu’s humidity index (HI) were obtained from 847 meteorological stations in China’s Three Northern Regions (1991–2020), sourced from the National Meteorological Science Data Center (https://data.cma.cn/). The dataset includes latitude, longitude, monthly mean temperature, and annual precipitation. To minimize errors in the results, 44 *Caragana* species with no fewer than five distribution records in China’s Three Northern Regions were retained. Using ArcGIS 10.8 software, the original moisture–temperature indexes calculated from formulas were spatially interpolated via the Ordinary Kriging method and converted into gridded data at 2.5 arc-minute resolution. The Ordinary Kriging method is suitable for addressing uneven distribution of meteorological stations and can quantify the uncertainty in interpolation results. And the cross-validation results show that the RMSE values for WI, CI, and HI are 11.09, 7.15, and 2.90, respectively. Multivariate linear regression equations were established using SPSS 20 software.

(1) WI=∑t−5

(2) CI=−∑5−t

HI = P/WI(3)

where WI represents the warmth index (°C·month), CI represents the coldness index (°C·month), HI represents the humidity index (mm/°C·month), t represents monthly mean temperature (°C), and P represents annual precipitation (mm).

When there is abundant species distribution data, the warmth index distribution curve exhibits a symmetrical normal distribution pattern [35]. The thermal distribution range was determined using the peak width at half height (PWHH) method, ensuring that approximately 78% of each species’ distribution falls within the optimal thermal range.

PWH = 2.354·S (4)

Optimal range = X − 0.5PWH~X + 0.5PWH(5)

where PWH represents the peak width at half height, S represents the standard deviation of the species’ warmth index values, and X represents the mean value of the species’ warmth index.

## 3. Results

### 3.1. Evaluation of Model Accuracy and Variable Importance

**Figure 2:**
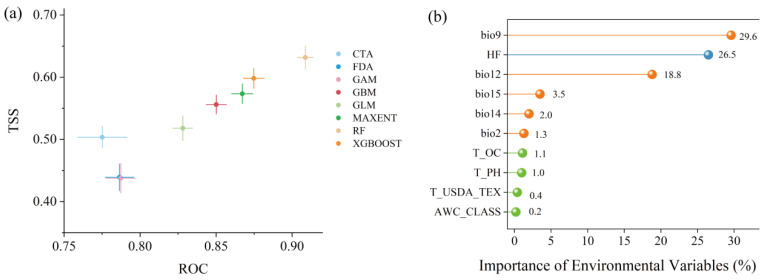
(**a**) The values of AUC and TSS in single models. (**b**) The importance of environmental variables in the ensemble model.

The TSS and AUC values of the eight different algorithms are shown in Figure 2a. The RF model exhibited the highest AUC and TSS scores. The RF model demonstrated superior performance compared to seven individual models due to its robustness against overfitting and exceptional capability to capture nonlinear relationships and complex interactions among environmental factors. Four well-performing single models (RF, XGBoost, MaxEnt, and GBM) with TSS values > 0.55 were selected for biomod2 ensemble construction. Compared to single models, the ensemble model employing the EMwmean approach showed significantly improved predictive accuracy, achieving AUC and TSS values of 0.93 and 0.69, respectively, indicating robust capability in projecting the habitat suitability of *Caragana* spp. Therefore, all subsequent analyses in this study are based on results derived from the ensemble model.

The importance of the screened current environmental variables was evaluated based on the ensemble model. The results in Figure 2b show that climatic and anthropogenic factors exert significantly stronger influences on genus *Caragana* distributions compared to soil variables. Among these, the mean temperature of the driest quarter (bio9) has the greatest impact, followed sequentially by the human footprint index (HF), annual precipitation (bio12), precipitation seasonality (bio15), and so on.

### 3.2. Current and Future Distribution of the Suitable Habitats of Caragana spp. in China’s Three Northern Regions

**Figure 3:**
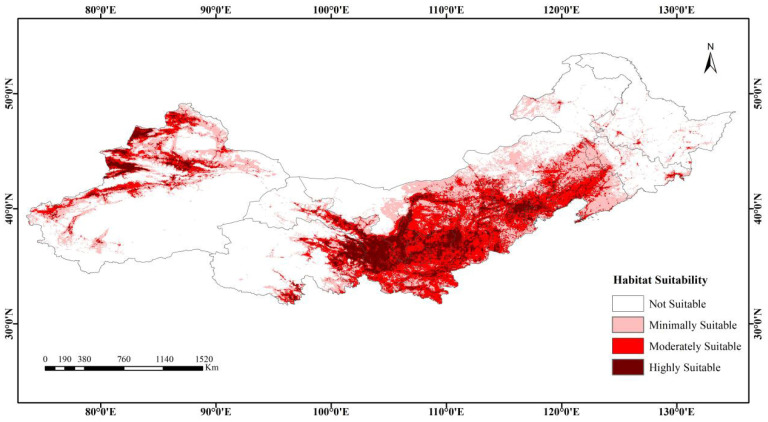
Potential distribution of *Caragana* spp. in China’s Three Northern Regions under the current climate scenario.

The projected current potential distribution of *Caragana* spp. is shown in Figure 3. Projections of suitable habitats under current climate scenarios closely match the actual distribution areas of *Caragana* species. These suitable habitats are primarily concentrated in southern Northeast China, central North China, and the eastern and western regions of Northwest China. All 13 provincial-level administrative regions are involved, but the primary distribution areas include southern Liaoning, southern Inner Mongolia, eastern Qinghai, central-eastern Gansu, mountainous western Xinjiang, Tianjin, Beijing, Hebei, Shanxi, Shaanxi, and Ningxia. The predicted potential habitat had a total area of 243.78 × 10^4^ km^2^, habitats classified as highly suitable, moderately suitable, and minimally suitable occupied areas of 45.29 × 10^4^ km^2^, 90.60 × 10^4^ km^2^, and 107.89 × 10^4^ km^2^, respectively, accounting for 7.97%, 15.94%, and 18.98% of the predicted habitat area.

**Figure 4:**
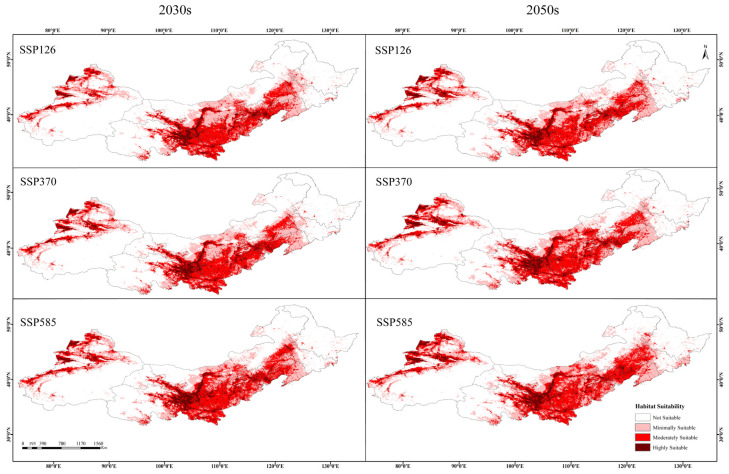
Potential distribution of *Caragana* spp. in China’s Three Northern Regions under different climate scenarios.

**Figure 5:**
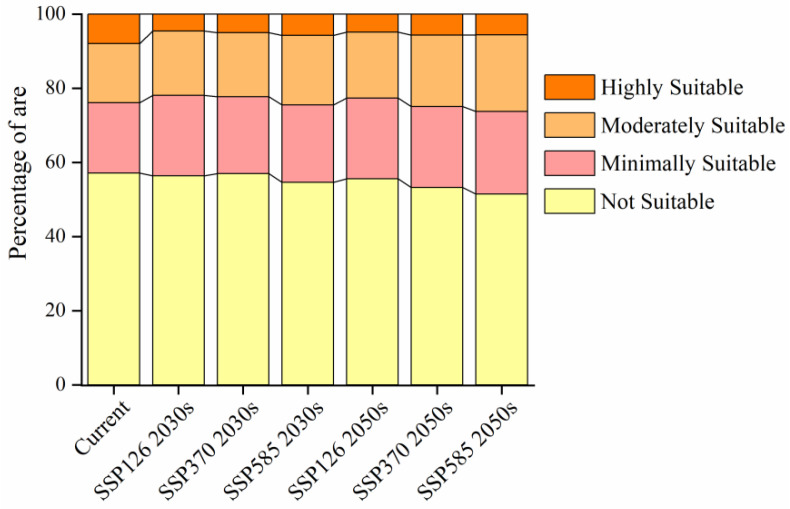
Changes in the area of suitable habitat for *Caragana* spp. in China’s Three Northern Regions under different climate scenarios.

Under future climate scenarios, the suitable habitat area for *Caragana* species in China’s Three Northern Regions exhibits an overall expansion trend (Figure 4), manifesting a corridor expansion phenomenon. And under future climate warming conditions, newly emerged *Caragana* suitable habitats appear in scattered pockets across western Gansu and Qinghai provinces, suggesting these high-altitude zones could serve as emerging refugia. Under the SSP126-2030s, SSP370-2030s, SSP585-2030s, SSP126-2050s, SSP370-2050s, and SSP585-2050s scenarios, the areas of suitable habitats are projected to be 247.96 × 10^4^ km^2^, 244.72 × 10^4^ km^2^, 258.00 × 10^4^ km^2^, 252.41 × 10^4^ km^2^, 266.11 × 10^4^ km^2^, and 275.83 × 10^4^ km^2^, accounting for 43.62%, 43.05%, 45.39%, 44.41%, 46.82%, and 48.53% of the total study area, respectively (Figure 5). Compared to the current climate scenario, highly suitable habitats under the same SSP scenarios decrease over time, while moderately and minimally suitable habitats increase in area.

### 3.3. Suitable Habitat Overlap and Centroid Migration

**Figure 6:**
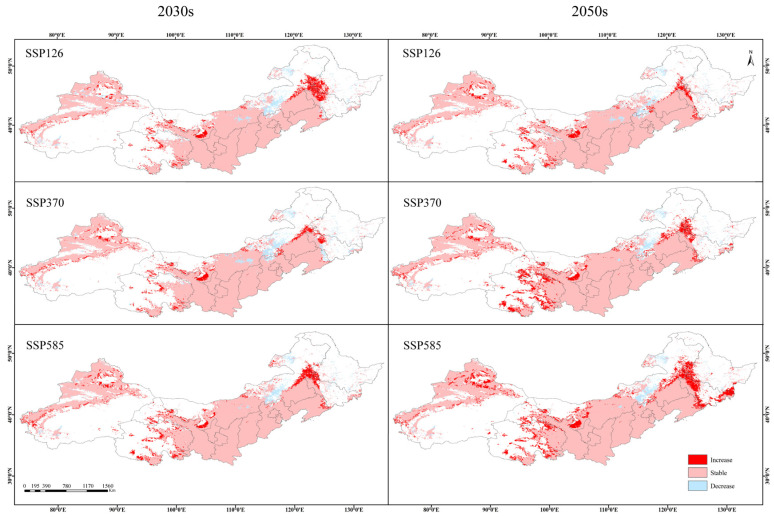
Spatial variations in the suitable habitat for *Caragana* spp. in China’s Three Northern Regions under different climate scenarios.

Compared to current climate scenario, the suitable distribution ranges of *Caragana* species expand outward under projected future climate scenarios in response to global warming (Figure 6). Divergent trends in habitat expansion emerge across different climate scenarios and geographic regions, while contraction zones are predominantly concentrated near the Hunshandake Sandy Land and Hailar River in Inner Mongolia. On the one hand, under the same SSP scenarios, as time goes on, the loss area of the suitable area gradually decreases, and the expansion area gradually increases. On the other hand, except in the ssp370-2030s scenario, at the same time, as the SSP scenario intensifies, the loss area of the suitable area gradually decreases, and the expansion area gradually increases, indicating that the high-emission scenario accelerates the spread of *Caragana* spp. to a wider area.

**Figure 7:**
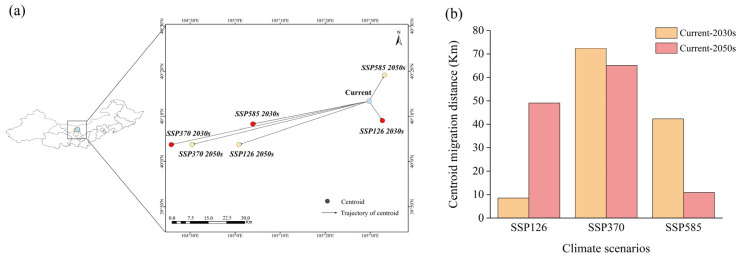
Changes in the center of mass of *Caragana* spp. in different climatic scenarios. (**a**) Centroid migration. (**b**) Centroid migration distance.

Regarding centroid migration (Figure 7), both current and future core distributions of *Caragana* spp. are centered in Alxa Left Banner, Inner Mongolia. Under the SSP126-2030s scenario, the centroid displacement is minimal, with a southeastern migration of 8.54 km. Conversely, under SSP370-2030s and SSP370-2050s scenarios, the maximum centroid migration occurs, showing southwestern migration of 72.41 km and 65.13 km, indicating stronger impacts of moderate-stress scenario on the dynamics of species distribution. In general, the distribution of *Caragana* species mainly shows a trend of migration to low latitudes under future climate scenarios.

### 3.4. Correlations Between the Geographic Distribution of Caragana Species in China’s Three Northern Regions and Climatic Factors

In order to further explore the relationship between *Caragana* species and moisture–temperature indexes in climatic conditions, a multiple linear regression equation was established with three-dimensional geographical elements (latitude, LAT; longitude, LONG; altitude, ALT) as independent variables and warmth index, WI; coldness index, CI; and humidity index, HI as dependent variables. The results are as follows

WI = 281.795 − 0.714LON − 2.288LAT − 0.025ALT (R^2^ = 0.657 *p* < 0.05)

CI = 257.092 − 0.492LON − 5.745LAT − 0.015ALT (R^2^ = 0.781 *p* < 0.05)

HI = −5.760 + 0.130LON − 0.187LAT + 0.004ALT (R^2^ = 0.444 *p* < 0.05)

where R^2^ is the coefficient of determination, and *p* is the significance of the coefficients for each one of the regressions.

The results show significant correlations between warmth index (WI), coldness index (CI), and humidity index (HI) with latitudinal, longitudinal, and elevational gradients of species distributions. In China’s Three Northern Regions, at constant elevation, per 1° northward latitude increase, WI decreases by 2.288 °C·month, CI reduces by 5.745 ℃·month, and HI declines by 0.187 mm/°C·month; per 1 °eastward longitude increase, WI decreases by 0.714 °C·month, CI reduces by 0.492 °C·month, and HI rises by 0.130 mm/°C·month; per 100 m elevation gain, WI decreases by 2.5 °C·month, CI reduces by 1.5 °C·month, and HI increases by 0.4 mm/°C·month.

The high sensitivity of both WI and CI to latitudinal gradients highlights the dual constraints imposed by growing-season thermal accumulation and overwintering extreme cold on *Caragana* species distribution patterns. This reflects *Caragana*’s climatic adaptation mechanisms and their role in defining the species’ geographic distribution limits.

### 3.5. Moisture–Temperature Indexes Distribution Ranges of Caragana Species in China’s Three Northern Regions

According to Formulas (1)–(5), the moisture–temperature indexes of 44 species of *Caragana* in China’s Three Northern Regions are calculated (Table 2). It can be seen from the results that different species show great differences in the demand for thermal energy. The average value of the WI of *Caragana* species is mostly in the range of 60–100 °C·month. However, a few species distributed at higher elevations, such as *C. chinghaiensis*, *C. junatovii*, *C. versicolor*, *C. erinacea*, *C. brevifolia*, and *C. densa*, show lower WI values (20–60 ℃·month). Conversely, a few species located in the south, such as *C. sinica*, *C. polourensis*, *C. stipitata*, *C. pekinensis,* and others, show high WI values, reaching more than 100 °C·month.

The majority of the Three Northern Regions of China experiences an arid climate, with the average humidity index of most *Caragana* species’ distribution areas ranging of 2–6 °C·month/mm, classifying these regions as arid to semi-arid. However, a few species such as *C. chinghaiensis*, *C. junatovii*, *C. versicolor*, and *C. erinacea* inhabit high-altitude shrublands with abundant precipitation, exhibiting significantly higher HI values exceeding 15 mm/°C·month.

### 3.6. Groups of Moisture–Temperature Distribution Caragana Species in China’s Three Northern Regions

According to Kira’s warmth index (WI) and Xu’s humidity index (HI), the 44 *Caragana* species in China’s Three Northern Regions were classified into five moisture–temperature distribution types, as shown in Table 3.

(1) Cold-Temperate Humid Type (20 ≤ WI < 60, H > 7.5). The climate is characterized by cold temperatures but relatively sufficient moisture availability. This type includes *C. brevifolia*, *C. chinghaiensis*, *C. densa*, *C. erinacea*, *C. junatovii*, *C. tangutica*, and *C. versicolor*. These species are characterized by high-altitude distributions, typical of alpine shrub communities. Except for a portion of *C. densa* distributed in the Tianshan Mountains of Xinjiang (which exhibits a disjunct distribution pattern), all other species within this type are confined to the mountain ranges of the northeastern marginal Qinghai–Tibet Plateau. Due to the cold and humid climate, the values of WI are very low, while the HI values are very high.
(2) Mesothermal Xeric Type (60 ≤ WI < 75, 3.5 ≤ HI ≤ 7.5). The climate features moderate temperatures but suboptimal moisture availability. This type includes *C. licentiana*, *C. opulens*, *C. pygmaea*, *C. stenophylla*, and *C. tibetica*. These species are predominantly distributed in China’s semi-arid regions, concentrated in eastern Gansu, Ningxia, and Inner Mongoli, exhibiting relatively high values of WI and low HI.
(3) Mesothermal Humid Type (60 ≤ WI < 75, HI > 7.5). The climate is characterized by moderate temperatures coupled with favorable moisture conditions. This type includes *C.jubata.* It is a typical discontinuous distribution species, which is distributed under the forests in the mountains of North China and Northwest China, separated by a vast arid grassland area in the middle. The humid habitat exhibits favorable thermal and moisture regimes, so the values of WI and HI are both higher.
(4) Warm-Temperate Hyperxeric Type (75 ≤ WI < 90, HI < 3.5). The climate features warm temperatures coupled with extreme aridity. This type includes *C. brachypoda*, *C. camilloi-schneideri*, *C. dasyphylla*, *C. kirghisorum*, *C. pleiophylla*, *C. polourensis*, *C. pruinosa*, *C. pumila*, *C. tragacanthoides,* and *C. turfanensis*. Most are desert species, primarily distributed in arid regions of China, inhabiting desert areas such as western Inner Mongolia, Ningxia, and Xinjiang. These areas experience favorable thermal conditions but arid climates, resulting in high WI values and very low HI values.
(5) Warm-Temperate Xeric Type (75 ≤ WI < 90, 3.5 ≤ HI ≤ 7.5). The climate is characterized by warm temperatures and limited moisture availability. This type contains the most species-rich group, accounting for 46% of all *Caragana* species. The genus *Caragana* exhibits a broad geographic distribution, spanning all provinces across China’s Three Northern Regions, yet its populations are most densely concentrated in the North China region. Compared to the Warm-Temperate Hyperxeric Type, this type exhibits a southeastern distributional shift, characterized by high WI values and low HI values. This type includes *C. acanthophylla*, *C. arborescens*, *C. aurantiaca*, *C. boisii*, *C. bongardiana*, *C. davazamcii*, *C. kansuensis*, *C. korshinskii*, *C. leucophloea*, *C. leveillei*, *C. liouana*, *C. microphylla*, *C. pekinensis*, *C. potaninii*, *C. purdomii*, *C. rosea*, *C. sinica*, *C. soongorica*, *C. stipitata*, *C. turkestanica,* and *C. zahlbruckneri.*

Based on moisture–temperature indexes, the Cold-Temperate Humid Type likely represents the most vulnerable among the five types. This type, dependent on cryo-humid environments, exhibits acute sensitivity to warming. And climate warming will lead to the contraction of high-altitude habitats. Under future climate warming scenarios, the distribution ranges of Mesothermal Xeric Type and Warm-Temperate Hyperxeric Type are projected to expand due to their thermal plasticity and drought-resilient traits, enabling them to displace hydric types in regions with exacerbated aridity.

## 4. Discussion

### 4.1. Current Suitable Habitats and Key Environmental Variables for Caragana Species

Prediction results indicate that the current suitable habitats of *Caragana* spp. exhibit geographic heterogeneity, mainly distributed in the transition zone from the northeast plain to the Mongolian Plateau, the Loess Plateau Ecological Region and the Northwest Arid Mountainous system, most of which belong to arid and semi-arid regions. The predicted distribution range exhibits strong spatial consistency with actual occurrence records. The ensemble model achieved AUC and TSS values of 0.93 and 0.69, respectively, further confirming its relatively high predictive accuracy. In terms of the importance of environmental variables, species distribution is influenced by the combined effects of climate, topography, soil, and human activities. However, at the macro-scale, climate is the dominant driver governing species distribution patterns [36,37]. Our findings confirm that climatic factors exert the strongest influence on current *Caragana* spp. habitat suitability, with the mean temperature of the driest quarter (bio9) and annual precipitation (bio12) playing pivotal roles. This aligns with Tu et al.’s conclusions, which similarly identified precipitation and temperature as the dominant climatic factors for *Caragana* species [38]. This reflects the genus’ exceptional adaptation to drought stress, which is mechanistically linked to its deep root systems, tolerance to extreme temperature fluctuations, and high-efficiency water-use strategies [39]. The most influential climatic factor is the bio9, which directly represents the thermal environment experienced by plants during the quarter of most severe moisture stress. The Three-North Region exhibits pronounced seasonal drought, particularly during the late spring to early summer transition period. During this period, *Caragana* spp. undergoes its primary biomass accumulation, which coincides with its seasonal growth pattern. Excessively low or high mean temperatures in the driest quarter will both adversely affect the growth and development of *Caragana* species. Human activities are the second most critical environmental factor affecting the *Caragana* species’ habitat suitability area. On one hand, areas highly affected by human activities—such as dense road networks, built-up zones, and agricultural expansion—can destroy *Caragana*’s natural dispersal corridors and lead to the loss of its original habitats. On the other hand, human activities like grazing and road transportation may facilitate seed dispersal (e.g., wind-mediated, animal-carried) [40]. Although soil factors showed limited explanatory power in our study, they may indirectly mediate *Caragana* spp. distribution through pathways such as the high permeability of sandy soils enhancing precipitation utilization efficiency [41], thus amplifying the effects of climatic drivers on the genus’ population dynamics.

### 4.2. Changes in the Suitable Habitats for Caragana spp. in the Future

This study uncovers the spatiotemporal dynamics of *Caragana* species distributions across in China’s Three Northern Regions under future climate change scenarios. The future climate scenarios will favor the growth and distribution of *Caragana*. This finding aligns with Cui et al.’s study on shrub expansion under climate warming [42]. Global warming and increased drought frequency will lower groundwater levels, thereby reducing the competitiveness of shallow-rooted herbaceous plants. In contrast, deep-rooted shrub species gain competitive advantages under these conditions. This competitive edge promotes shrub growth, establishment, and abundance, ultimately driving grassland-to-shrubland transitions [43]. Notably, the contraction of suitable habitats for *Caragana* spp. is predominantly concentrated near the Hunshandake Sandy Land and Hailar River in Inner Mongolia, potentially linked to localized climate change-induced aridity intensification or precipitation regime shifts [44]. The contraction of highly suitable habitats and expansion of minimally and moderately suitable habitats suggest that *Caragana* spp. may broaden its tolerance range through niche differentiation strategies in response to climate warming-driven environmental filtering pressures [45]. Niche differentiation strategies comprise intraspecific genetic variation and phenotypic plasticity. This discovery challenges the assumption of ‘homogenized habitat suitability response’ in conventional species distribution models (SDMs), aligning with the perspective of Dallas et al. [46]. The distribution centroids exhibited an overall poleward shift, consistent with the expansion of climatic zones in arid and semi-arid regions under global warming [47]. *Caragana* species, mostly shrubs with limited dispersal capacity, typically exhibit seed dispersal within a hundred-meter scale. When centroid shifts substantially exceed natural dispersal distances, this not only reflects climatic pressure intensity but also reveals the species’ viability threshold [48]. The minimal centroid displacement under SSP126 reflects relative habitat stability under low climate stress. Under the SSP370 scenario, *Caragana* species exhibit greater displacement than under SSP585, which could be influenced by nonlinear climatic factors and the species’ tolerance threshold. Under SSP370, intense aridification in specific regions forces *Caragana* species into massive coherent migration, resulting in long-distance centroid displacement. Conversely, under SSP585, extreme climatic pressures may cause large-scale contraction of original habitats, with populations contracting into fragmented “refugia” near their native distribution—resulting in relatively smaller calculated centroid displacement distances.

### 4.3. Zonal Distribution of Caragana Species in the Three Northern Regions of China 

**Figure 8:**
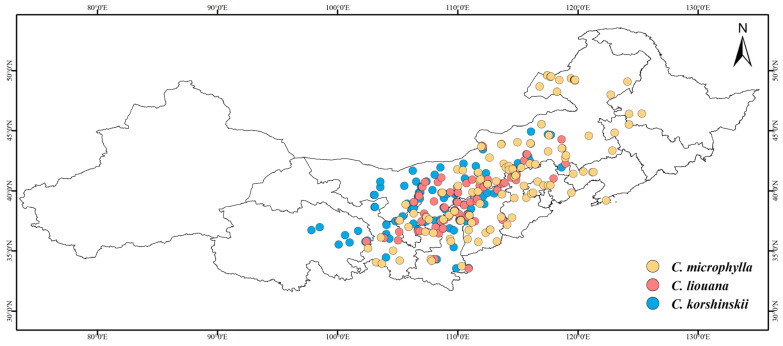
A band-like replacement pattern of *C. microphylla, C. liouana*, and *C. korshinskii*.

Studies have shown that the distribution of *Caragana* species exhibits a zonational pattern consistent with climatic conditions, where moisture and temperature play pivotal roles [49]. However, previous studies on the zonal distribution patterns of *Caragana* species have predominantly focused on qualitative descriptions, lacking specific quantitative analyses. Interestingly, our calculations based on moisture–temperature indexes revealed distinct zonal and replacement distribution patterns among some *Caragana* species. For instance, *C. microphylla*, *C. liouana*, and *C. korshinskii* exhibit a band-like replacement from east to west across steppe, desert-steppe, and desert zones, respectively (Figure 8). Their distribution patterns can be explained by humidity indices, with recorded mean values of 5.6, 5.0, and 4.8, respectively. According to Xu’s HI classification, they are distributed in semi-humid regions (5.5–7.5) and semi-arid regions (3.5–5.5), which aligns with results from preceding studies. In addition, this study synthesizes the zonal distribution of *Caragana* species into the following three points: (1) Species richness decreases with increasing precipitation and temperature but increases with decreasing precipitation and rising elevation [50]. The genus exhibits relatively low diversity in forest zones, yet gradually increases across steppe to desert zones. This reflects *Caragana*’s relatively strong adaptive capacity to drought-stressed environments. (2) Species occur across humid, semi-humid, semi-arid, and arid regions, each adapted to distinct climatic zones. This reflects that the genus is not confined to a single environment, but exhibits a broad ecological amplitude, thereby occupying distinct geographic spaces. (3) Driven by zonal environmental gradients, different species display sequential distribution patterns within specific geographic sectors. This spatial pattern is the result of the species’ long-term adaptation and differentiation to environmental gradients such as climate, forming a spatial sequence that closely corresponds to environmental conditions.

### 4.4. Prospective Research Priorities and Directions

This study elucidates the ecogeographic distribution patterns of *Caragana* species in China’s Three Northern Regions, providing a robust theoretical foundation for their ecological risk assessment, adaptive management, and biodiversity conservation and restoration [51,52]. However, this study also has some limitations, due to inherent constraints [53]. Firstly, both species occurrence records and environmental variables are obtained from online databases without field-collected data, potentially affecting accuracy. This may lead to model underestimation of ecological niches for marginal populations or microhabitat-specific species [54], which could explain the relatively lower TSS compared to AUC in model evaluations. Secondly, climate data used for species distribution modeling and moisture–temperature indexes calculations are sourced from different platforms with temporal discrepancies (1970–2000 vs. 1990–2020), potentially compromising analytical consistency. Additionally, the moisture–temperature indexes for the Three Northern Regions of China were interpolated from 847 meteorological stations, with sparse coverage in environmentally harsh areas inevitably introducing spatial estimation errors. Finally, our regional-scale moisture–temperature indexes only considered horizontal climatic gradients, neglecting vertical (elevational) distributional variations in species. Therefore, future similar research should overcome these problems. To address these limitations, future efforts will supplement field sampling data, focusing on marginal populations and microhabitat-specific species distribution areas, while integrating remote sensing technology with community surveys to improve distribution point precision. Additionally, we will further develop a topographic-climate coupled model to quantify the effects of moisture–temperature redistribution along vertical gradients, achieving a three-dimensional ecological niche simulation.

## 5. Conclusions

In this study, we first employed the Biomod2 ensemble model to project current and future climate scenario-based distributions of *Caragana* spp. in China’s Three Northern Regions, incorporating both natural and anthropogenic drivers. Subsequently, to further analyze species-specific hydrothermal relationships, we calculated Kira’s warmth index (WI), coldness index (CI), and Xu’s humidity index (HI) for 44 *Caragana* species across the study area. The results show the following: (1) The mean temperature of the driest quarter (bio9), human footprint index (HF), and annual precipitation (bio12) are the key environmental drivers of current *Caragana* spp. distributions. (2) *Caragana* species are mainly distributed in the transition zone from the northeast plain to the Mongolian Plateau, the Loess Plateau Ecological Region and the Northwest Arid Mountainous system, most of which are classified as arid and semi-arid regions. (3) Under future climate scenarios, the suitable habitats of *Caragana* spp. are projected to expand outward in response to global warming. Additionally, the majority of centroids will shift toward lower latitudes. High-stress scenarios induce significantly greater changes in suitable areas compared to low-stress conditions. (4) *Caragana* species exhibit substantial interspecific variation in hydrothermal requirements. Significant correlations exist between WI, CI, and HI values and species distribution patterns across latitudinal, longitudinal, and elevational gradients. Integrating WI and HI enables classification of the 44 *Caragana* species in China’s Three Northern Regions into five distinct types based on Kira–Xu’s moisture–temperature indexes. The research findings will provide critical practical guidance for biodiversity conservation of *Caragana* species, the Three-North Shelterbelt Program and related ecological projects, as well as the restoration and management of degraded ecosystems in arid and semi-arid regions under global climate change.

## Figures and Tables

**Table 1:** Environmental variables involved in the prediction modeling of *Caragana*.

Datasets	Variables	Description	Units
Bioclimatic variables	bio2	Mean diurnal range (mean of monthly (max temp–min temp))	°C
	bio9	Mean temperature of driest quarter	°C
	bio12	Annual precipitation	mm
	bio14	Precipitation of driest month	mm
	bio15	Precipitation seasonality (coefficient of variation)	—
Human activities	HF	The human footprint index	—
Topsoil variables	T_OC	Topsoil organic carbon	% weight
	T_pH	Topsoil pH (H_2_O)	−log(H^+^)
	T_USDA_TEX	Topsoil USDA texture classification	name
	AWC_CLASS	Available water content class	code

**Table 2:** The moisture–temperature indexes of *Caragana* species in China’s Three Northern Regions.

No.	Type	Species	Frequency	WI				CI			HI		
				Full Range	Optimal Range	Mean	S.D.	Full Range	Mean	S.D.	Full Range	Mean	S.D.
1	(5)	***C. acanthophylla***	50	72.1~113.6	76.9~97.7	87.3	8.8	−106.6~−40.0	−58.7	9.1	0.9~12.2	6.3	3.1
2	(5)	*C. arborescens*	157	36.3~118.9	66.2~108.1	87.1	17.8	−143.1~−2.7	−48.8	30.2	2.0~12.0	6.2	2.0
3	(5)	*C. aurantiaca*	22	27.2~114.5	64.7~113.2	88.9	20.6	−78.9~−32.3	−47.8	10.9	0.6~17.4	3.8	4.4
4	(5)	*C. boisii*	7	76.3~117.8	78.4~119.8	99.1	17.6	−40.3~−3.4	−16.8	14.1	2.0~8.1	5.9	2.2
5	(5)	*C. bongardiana*	8	77.2~91.4	77.4~87.6	82.5	4.4	−70.1~−57.9	−63.2	4.2	2.1~11.3	5.4	3.6
6	(4)	***C. brachypoda***	48	60.3~92.4	67.8~92.6	80.2	10.5	−92.2~−26.1	−45.8	16.3	1.6~9.8	3.3	1.8
7	(1)	*C. brevifolia*	104	17.0~84.9	29.6~59.3	44.5	12.6	−80.4~−12.8	−45.5	12.5	3.9~44.5	14.1	9.0
8	(4)	***C. camilloi-schneideri***	10	75.9~97.0	77.4~97.4	87.4	8.5	−77.0~−49.5	−58.9	10.5	1.5~3.7	2.5	0.9
9	(1)	*C. chinghaiensis*	32	16.9~42.7	17.4~30.0	23.7	5.4	−87.1~−37.4	−67.1	12.1	9.5~52.0	29.1	12.0
10	(4)	***C. dasyphylla***	12	87.6~111.4	93.8~109.3	101.5	6.6	−65.6~−23.5	−33.4	10.8	0.4~6.5	1.9	1.6
11	(5)	*C. davazamcii*	5	72.9~89.9	74.1~90.9	82.5	7.1	−40.6~−29.4	−34.9	4.8	2.3~6.1	4.6	1.6
12	(1)	*C. densa*	35	18.9~101.5	28.8~81.1	55.0	22.2	−109.9~−13.3	−44.1	18.5	0.4~44.8	12.9	9.9
13	(1)	*C. erinacea*	46	15.7~83.6	13.2~53.8	33.5	17.3	−81.0~−8.4	−50.0	15.6	2.6~45.6	21.8	10.6
14	(3)	*C. jubata*	104	15.8~116.3	28.4~102.5	65.5	31.5	−80.4~−11.2	−43.8	14.0	0.7~44.5	10.7	9.8
15	(1)	*C. junatovii*	10	20.5~26.9	21.1~27.1	24.1	2.6	−70.7~−39.5	−55.5	11.7	17.6~29.2	22.6	4.5
16	(5)	*C. kansuensis*	56	64.5~110.1	70.2~93.0	81.6	9.7	−48.3~−14.3	−31.5	6.2	2.0~8.0	4.9	1.5
17	(4)	***C. kirghisorum***	6	90.4~96.9	91.6~97.4	94.5	2.5	−59.5~−50.0	−53.2	4.2	1.5~3.5	2.2	0.8
18	(5)	***C. korshinskii***	115	33.1~119.4	60.6~96.8	78.7	15.4	−91.4~−3.3	−44.1	17.9	0.8~14.9	4.8	2.5
19	(5)	***C. leucophloea***	112	48.5~126.8	69.0~100.2	84.6	13.3	−94.2~−23.5	−55.0	13.9	0.1~14.2	3.7	2.9
20	(5)	*C. leveillei*	40	83.5~122.0	87.6~114.6	101.1	11.5	−43.4~−1.9	−20.5	10.4	4.1~9.3	5.6	1.0
21	(2)	***C. licentiana***	44	32.0~91.6	51.4~83.2	67.3	13.5	−57.1~−16.7	−35.0	7.6	1.5~22.7	7.1	4.4
22	(5)	*C. liouana*	81	43.7~113.6	67.3~93.6	80.4	11.2	−120.1~−5.0	−44.1	16.9	1.6~14.2	5.0	1.8
23	(5)	*C. microphylla*	103	32.0~115.2	59.0~100.5	79.8	17.6	−120.1~−6.2	−52.2	29.6	2.1~22.7	5.6	2.3
24	(2)	***C. opulens***	169	22.9~112.7	46.1~92.9	69.5	19.9	−73.2~−5.1	−38.6	13.4	1.7~36.2	7.3	5.0
25	(5)	*C. pekinensis*	56	89.9~122.0	102.3~115.3	108.8	5.5	−43.9~−12.0	−24.5	5.3	4.1~6.0	5.1	0.4
26	(4)	***C. pleiophylla***	18	84.6~106.2	91.1~106.0	98.5	6.3	−66.7~−26.5	−38.3	11.5	0.4~7.6	1.9	1.8
27	(4)	***C. polourensis***	46	92.8~114.1	98.1~115.0	106.6	7.1	−52.8~−20.9	−28.2	6.4	0.2~3.1	1.1	0.9
28	(5)	*C. potaninii*	6	82.6~97.8	81.6~96.3	89.0	6.3	−44.2~−19.6	−34.7	9.9	4.6~6.6	5.3	0.8
29	(4)	***C. pruinosa***	18	73.8~113.6	83.4~110.4	96.9	11.5	−79.2~−26.5	−42.3	17.0	0.2~5.8	1.9	1.3
30	(4)	***C. pumila***	43	74.1~122.7	74.4~101.9	88.2	11.7	−82.3~−33.2	−57.6	11.5	0.6~10.1	3.3	2.4
31	(5)	*C. purdomii*	72	81.5~121.5	81.9~103.8	92.8	9.3	−45.4~−2.0	−26.5	9.2	1.7~9.0	5.7	1.2
32	(2)	***C. pygmaea***	50	58.8~104.2	58.5~84.3	71.4	11.0	−92.3~−24.2	−63.6	19.4	2.3~9.3	4.3	1.6
33	(5)	*C. rosea*	150	43.9~123.2	82.1~114.9	98.5	13.9	−62.0~−0.9	−28.2	11.9	3.3~15.2	5.8	1.4
34	(5)	*C. sinica*	94	49.7~122.5	86.5~121.5	104.0	14.9	−50.1~−1.2	−17.9	13.0	4.1~10.6	6.5	1.8
35	(5)	***C. soongorica***	23	76.9~113.6	79.6~98.1	88.8	7.9	−94.6~−43.2	−64.1	11.2	1.4~9.9	4.9	2.7
36	(2)	***C. stenophylla***	166	28.7~99.4	59.9~90.0	75.0	12.8	−128.3~−23.9	−55.9	25.1	1.4~15.2	4.0	2.0
37	(5)	*C. stipitata*	50	83.5~121.9	97.5~116.1	106.8	7.9	−33.9~−3.4	−13.6	7.5	4.1~9.3	7.1	1.3
38	(1)	*C. tangutica*	31	23.1~83.0	40.6~78.1	59.3	15.9	−49.7~−15.5	−32.4	10.8	2.9~27.7	8.6	6.0
39	(2)	***C. tibetica***	62	24.1~92.4	43.4~94.5	69.0	21.7	−68.7~−26.5	−41.2	10.7	1.7~22.9	6.4	5.8
40	(4)	***C. tragacanthoides***	11	73.1~84.8	73.3~84.0	78.7	4.5	−76.7~−61.8	−66.5	4.2	1.9~2.8	2.4	0.3
41	(4)	***C. turfanensis***	17	87.6~111.4	94.1~107.8	100.9	5.8	−65.6~−23.5	−35.2	10.0	0.7~6.5	1.8	1.4
42	(5)	***C. turkestanica***	8	73.8~106.2	71.4~98.7	85.0	11.6	−100.7~−26.5	−63.3	21.0	1.3~11.1	5.0	3.8
43	(1)	*C. versicolor*	9	16.3~58.8	12.5~53.7	33.1	17.5	−79.8~−35.1	−58.8	18.4	7.6~43.2	24.7	15.6
44	(5)	*C. zahlbruckneri*	49	65.1~115.2	80.7~111.2	95.9	13.0	−66.4~−15.2	−34.7	11.7	4.3~7.5	5.5	0.8

The symbols in the “Type”column represent the moisture–temperature distribution type for the corresponding *Caragana* species (see Section 3.6 for details). And in the “Species” column, boldfaced entries represent *Caragana* species well adapted to arid regions.

**Table 3:** Groups of moisture–temperature distribution *Caragana* species.

	(Kira’s WI-Xu’s HI)				
	Class of HI (mm/°C·Month)				Total
		<3.5	3.5~7.5	>7.5	
	20~60			7 * 9 12 13 15 38 43	7
Class of WI (°C·month)	60~75		21 24 32 36 39	14	6
	75~90	6 8 10 17 26 27 29 30 40 41	1 2 3 4 5 11 16 18 19 20 22 23 25 28 31 33 34 35 37 42 44		31
Total		10	26	8	44

The asterisk (*) denotes the species number; details are provided in Table 2.

## Data Availability

The original contributions presented in this study are included in the article. Further inquiries can be directed to the corresponding author.

## Supplementary Materials

The following supporting information can be downloaded at: https://www.mdpi.com/article/10.3390/plants14152368/s1.
