# Supplementary material for: Unveiling the Impact of Climatic Factors on the Distribution Patterns of Caragana spp. in China’s Three Northern Regions
Source: Plants (Basel). 2025 Aug 1;14(15):2368. doi: 10.3390/plants14152368 (PMC12349240; doi:10.3390/plants14152368)
Supplement: Supplementary file 1 [file plants-14-02368-s001.zip › plants-3700727-supplementary.pdf]

Supplementary materials

(44 *Caragana* species occurrences information from GBIF)

1. GBIF.org (21 November 2024) GBIF Occurrence Download https://doi.org/10.15468/dl.pzhxwn
2. GBIF.org (21 November 2024) GBIF Occurrence Download https://doi.org/10.15468/dl.c7g6xp
3. GBIF.org (23 November 2024) GBIF Occurrence Download https://doi.org/10.15468/dl.b7ajjt
4. GBIF.org (23 November 2024) GBIF Occurrence Download https://doi.org/10.15468/dl.bahtb4
5. GBIF.org (23 November 2024) GBIF Occurrence Download https://doi.org/10.15468/dl.pd8f59
6. GBIF.org (21 November 2024) GBIF Occurrence Download https://doi.org/10.15468/dl.skj8zb
7. GBIF.org (23 November 2024) GBIF Occurrence Download https://doi.org/10.15468/dl.erfvuv
8. GBIF.org (23 November 2024) GBIF Occurrence Download https://doi.org/10.15468/dl.z2sbjq
9. GBIF.org (23 November 2024) GBIF Occurrence Download https://doi.org/10.15468/dl.pghsed
10. GBIF.org (23 November 2024) GBIF Occurrence Download https://doi.org/10.15468/dl.9pyfbq
11. GBIF.org (23 November 2024) GBIF Occurrence Download https://doi.org/10.15468/dl.f5zu6r
12. GBIF.org (21 November 2024) GBIF Occurrence Download https://doi.org/10.15468/dl.3kv4kg
13. GBIF.org (23 November 2024) GBIF Occurrence Download https://doi.org/10.15468/dl.te56pz
14. GBIF.org (19 November 2024) GBIF Occurrence Download https://doi.org/10.15468/dl.qa76gm
15. GBIF.org (05 March 2025) GBIF Occurrence Download https://doi.org/10.15468/dl.9fudgn
16. GBIF.org(23 November 2024) GBIF Occurrence Download https://doi.org/10.15468/dl.knmu7k
17. GBIF.org (23 November 2024) GBIF Occurrence Download https://doi.org/10.15468/dl.sncadt
18. GBIF.org (21 November 2024) GBIF Occurrence Download https://doi.org/10.15468/dl.9arhr9
19. GBIF.org (23 November 2024) GBIF Occurrence Download https://doi.org/10.15468/dl.85mets
20. GBIF.org (21 November 2024) GBIF Occurrence Download https://doi.org/10.15468/dl.y5xe63
21. GBIF.org (21 November 2024) GBIF Occurrence Download https://doi.org/10.15468/dl.zbtrnw
22. GBIF.org (21 November 2024) GBIF Occurrence Download https://doi.org/10.15468/dl.an7dav
23. GBIF.org (21 November 2024) GBIF Occurrence Download https://doi.org/10.15468/dl.p49zyq
24. GBIF.org (21 November 2024) GBIF Occurrence Download https://doi.org/10.15468/dl.nuj49h
25. GBIF.org(23 November 2024) GBIF Occurrence Download https://doi.org/10.15468/dl.5wafym
26. GBIF.org (23 November 2024) GBIF Occurrence Download https://doi.org/10.15468/dl.fc5gf2

27. GBIF.org (23 November 2024) GBIF Occurrence Download https://doi.org/10.15468/dl.jd3f2w

28. GBIF.org (23 November 2024) GBIF Occurrence Download https://doi.org/10.15468/dl.d24bez

29. GBIF.org (23 November 2024) GBIF Occurrence Download https://doi.org/10.15468/dl.uhfjda

30. GBIF.org (23 November 2024) GBIF Occurrence Download https://doi.org/10.15468/dl.sz6g3a

31. GBIF.org (23 November 2024) GBIF Occurrence Download https://doi.org/10.15468/dl.fpqz88

32. GBIF.org (21 November 2024) GBIF Occurrence Download https://doi.org/10.15468/dl.2naqf7

33. GBIF.org (21 November 2024) GBIF Occurrence Download https://doi.org/10.15468/dl.qajrhh

34. GBIF.org (21 November 2024) GBIF Occurrence Download https://doi.org/10.15468/dl.y5sc99

35. GBIF.org (23 November 2024) GBIF Occurrence Download https://doi.org/10.15468/dl.ugjjqy

36. GBIF.org (23 November 2024) GBIF Occurrence Download https://doi.org/10.15468/dl.4wk86k

37. GBIF.org (23 November 2024) GBIF Occurrence Download https://doi.org/10.15468/dl.gwya3f

38. GBIF.org(23 November 2024) GBIF Occurrence Download https://doi.org/10.15468/dl.dvnwws

39. GBIF.org (23 November 2024) GBIF Occurrence Download https://doi.org/10.15468/dl.z25y98

40. GBIF.org (23 November 2024) GBIF Occurrence Download https://doi.org/10.15468/dl.ng5y7w

41. GBIF.org (22 March 2025) GBIF Occurrence Download https://doi.org/10.15468/dl.g6encd

42. GBIF.org (23 November 2024) GBIF Occurrence Download https://doi.org/10.15468/dl.p4fvdq

43. GBIF.org (23 November 2024) GBIF Occurrence Download https://doi.org/10.15468/dl.g4na7e

44. GBIF.org (23 November 2024) GBIF Occurrence Download https://doi.org/10.15468/dl.u3zjjt
